# Supplementary material for: ScanFold 2.0: a rapid approach for identifying potential structured RNA targets in genomes and transcriptomes
Source: PeerJ. 2022 Nov 8;10:e14361. doi: 10.7717/peerj.14361 (PMC9651051; doi:10.7717/peerj.14361)
Supplement: Supplemental Information 7 — Each cell in the table is the percent difference in percent of predicted nucleotides that are paired. Positive values indicate mono- predicted more pairs and negative values indicate that di- predicted more pairs for the respective genome, version of SF, randomizations, and shuffling method. From left to right SF1 100 randomization difference in percent paired for -2 z-score pairs, SF1 1000 randomization difference in percent paired for -2 z-score pairs, SF1 10000 randomization difference in percent paired for -2 z-score pairs, SF2 difference in percent paired for -2 z-score pairs, SF1 100 randomization difference in percent paired for -1 z-score pairs, SF1 1000 randomization difference in percent paired for -1 z-score pairs, SF1 10000 randomization difference in percent paired for -1 z-score pairs, SF2 difference in percent paired for -1 z-score pairs, SF1 100 randomization difference in percent paired for no filter z-score pairs,SF1 1000 randomization difference in percent paired for no filter z-score pairs, SF1 10000 randomization difference in percent paired for no filter z-score pairs, and SF2 difference in percent paired for no filter z-score pairs. From top to bottom ZIKA, HIV-1, SARS-CoV-2 genomes that coincide with the differences in percent paired nucleotides. [file peerj-10-14361-s007.docx]

**Table S1.** **Percent difference in percent of predicted paried nucleotides comparing SF1 to SF1 mono and dinucleotide shuffling and SF2 to SF2 mono and dinucleotide shuffling**

|  | SF1 100  Mono vs di -2 | SF1  1000  Mono vs di -2 | SF1 10000 mono vs di -2 | SF2 mono vs di -2 | SF1 100 mono vs di -1 | SF1 1000 mono vs di -1 | SF1 10000 mono vs di -1 | SF2 mono vs di -1 | SF1 100 mono vs di No Filter | SF1 1000 mono vs di No Filter | SF1 10000 mono vs di No Filter | SF2 mono vs di No Filter |
| --- | --- | --- | --- | --- | --- | --- | --- | --- | --- | --- | --- | --- |
| ZIKA | 1.70 | 2.19 | 2.30 | 0.76 | 8.65 | 8.21 | 8.26 | 3.13 | -0.72 | -1.11 | -0.85 | -0.11 |
| HIV-1 | 1.72 | 1.88 | 1.81 | -1.46 | 4.06 | 4.06 | 3.92 | -1.66 | 0.44 | 0.87 | 0.78 | -1.13 |
| SARS-CoV-2 | 5.73 | 5.52 | 5.55 | -0.72 | 6.07 | 6.13 | 6.13 | 1.54 | 1.26 | 1.41 | 1.45 | 0.36 |

Positive values indicate mononucleotide shuffling predicted more pairs and negative values indicate that dinucleotide shuffling predicted more pairs for the respective genome, version of SF, randomizations, and shuffling method.
