# Supplementary material for: ScanFold 2.0: a rapid approach for identifying potential structured RNA targets in genomes and transcriptomes
Source: PeerJ. 2022 Nov 8;10:e14361. doi: 10.7717/peerj.14361 (PMC9651051; doi:10.7717/peerj.14361)
Supplement: Supplemental Information 8 — The average z-scores for all windows in every ScanFold run were determined and the average difference between shuffling techniques was calculated from all analyzed genomes. From left to right SF1 using 100 randomization mono-, SF1 using 100 randomizations di-, difference between SF1 using 100 randomizations with mono- and dinucleotide, SF1 using 1000 randomizations with mono-, SF1 using 1000 randomizations with di-, difference between SF1 using 1000 randomizations with mono- and dinucleotide, SF1 using 10000 randomizations with mono, SF1 using 10000 randomizations with di-, difference between SF1 using 10000 randomizations with mono- and dinucleotide, SF2 mono-, SF2 di-, and difference between SF2 with mono- and dinucleotide. From top to bottom ZIKA, HIV, SARS-CoV-2, and the average difference in z-score between shuffling techniques. [file peerj-10-14361-s008.docx]

**Table S2. Average z-scores from all ScanFold analysis windows for SF1 and SF2 using both shuffling methods and different randomizations**

The average z-scores for all windows in every ScanFold run were determined and the average difference between shuffling techniques was calculated from all analyzed genomes.

|  | **SF1 Mono 100** | **SF1 Di 100** | **SF1 100 Mono-Di** | **SF1 Mono 1000** | **SF1 Di 1000** | **SF1 1000 Mono-Di** | **SF1 Mono 10000** | **SF1 Di 10000** | **SF1 10000 Mono-Di** | **SF2 Mono** | **SF2 Di** | **SF2 Mono-Di** |
| --- | --- | --- | --- | --- | --- | --- | --- | --- | --- | --- | --- | --- |
| **Zika** | -0.645 | -0.235 | -0.411 | -0.649 | -0.234 | -0.414 | -0.649 | -0.234 | -0.415 | -0.892 | -0.731 | -0.161 |
| **HIV** | -0.455 | -0.137 | -0.318 | -0.465 | -0.140 | -0.326 | -0.467 | -0.141 | -0.326 | -0.638 | -0.746 | 0.108 |
| **SARS-CoV-2** | -1.499 | -1.147 | -0.351 | -1.522 | -1.167 | -0.355 | -1.525 | -1.170 | -0.356 | -1.686 | -1.681 | -0.005 |
| **Average Difference** |  |  | -0.360 |  |  | -0.365 |  |  | -0.366 |  |  | -0.019 |
